# Study of the Avoidable Mortality in Iran: Kerman Province

**DOI:** 10.5812/ircmj.9524

**Published:** 2013-04-05

**Authors:** Mohammadreza Amiresmaili, Narges Khanjani, Mahmood Nekoei Moghadam, Parvaneh Isfahani

**Affiliations:** 1 Research Center for Medical Informatics, Institute for Future Studies in Health, Kerman University of Medical Sciences, Kerman, IR Iran; 2 Department of Epidemiology and Biostatistics, School of Public Health, Kerman University of Medical Sciences, Kerman, IR Iran; 3 Department of Health Services Administration, Tehran University of Medical Sciences, Tehran, IR Iran

**Keywords:** Mortality, Hospitals, Iran

## Abstract

**Background:**

Recognizing mortality pattern and observing its trend will help us in determining health priorities, allocating health resources and priorities in health section, eliminating main factors of premature deaths and carrying out epidemiological research.

**Objective:**

The aim of this research was to determine avoidable mortality in Kerman province.

**Materials and Methods:**

The present study was carried out longitudinal. A checklist was applied for data collection. Avoidable mortalities were examined in Kerman province between 2004 and 2010. Statistical universe of this research was all deaths registered in statistical unit of health deputy of Kerman University of Medical Sciences; they were all studied through a census method. Data analysis was carried out using descriptive and analytical test by SPSS18.0.

**Results:**

Two thousand and one hundred –ninety seven deaths were examined. Of them, 210 deaths were unavoidable and 19987 were avoidable. In this study, most unavoidable deaths were due to pancreases cancer and ovary cancer.

**Conclusions:**

Relative high number of avoidable deaths indicates that performance of health system was not desirable in Kerman during the studied years. Examining trend of avoidable deaths showed that these mortalities are increasing although it is not significant nowadays. However, if this trend continues, it will be very alarming for health care authorities in this region.

## 1. Background

Different criteria are commonly used to evaluate the performance of health care system around the world. Most of these criteria focus on economic efficiency, usefulness of medical effects, social acceptability and social structure. Since suitable information is not available, consequences of health care are rarely evaluated. Moreover, the relationship between health results and health care are mainly vague under the influence of genetic, social and environmental factors; it affects health consequences according to health care. Existing evaluations about consequences of health care generally focus on performance of hospital and physicians; they contradict the general health or other health care systems (1). Over the last decades, different approaches have been employed to quantify the share of health services in peoples' health. One of the concepts mostly used in this case is mortality resulted from special reasons which shouldn't occur if services are provided effectively and on time. These types of deaths are called avoidable mortality or mortality amenable to medical/health care. This concept was first introduced by Rutstein et al. in 1976 (2). He introduced the concept of premature and unnecessary mortality as a new way to measure quality of medical care. For example, it includes infants and mothers' mortality and mortality resulted from diseases like tuberculosis, diabetes, influenza, and appendicitis and breast cancer (3). Avoidable mortalities through treatment and prevention (e.g. breast cancer, colon cancer, stomach cancer, duodenal ulcer, diabetes and hypertension) which happen before the age of 75 and are avoidable through medical interventions are called avoidable mortalities through treatment; those deaths which happen before the age of 75 and can be prevented through changes in lifestyles and general health policies are called avoidable mortality through prevention (e.g. vaccination, lung cancer, hepatitis B, burn, psychoactive drug abuse, AIDS and accidents. However, those types of deaths which are not avoidable using preventive measures and medical interventions (e.g. prostate cancer, pancreatic cancer and ovarian cancer) are called unavoidable mortalities (4). In his research, Nolte (2012) examined avoidable mortality in Australia and showed that risky health behaviors (smoking, lack of exercise) and prevalence of high risk factors (obesity) in low-income groups can increase avoidable mortality (5). Results of a study by Piers also revealed that the highest rate of avoidable mortality was related to male and female villagers who had the lowest access to health services and who were in low socioeconomic conditions (6). In his study, Bautista examined the effect of social factors on avoidable mortality in 2005 and showed that risk of avoidable mortality was higher in patients with low or middle education level; it was also shown that those patients who stayed for a long time in hospitals had lower risk of avoidable mortality (7). In his study, Mackenbach examined the relationship between avoidable mortality and health services in 1990 and showed that socioeconomic status and health care resources are effective in increasing the rate of avoidable mortality through treatment (8). Recognizing pattern and causes of mortality and preventing its risk factors is one of the most suitable guidelines to increase life expectancy. To provide people with a long life, mortality pattern and its causes in society must be recognized and prevented. Valid information about causes of death, description of death trend and its changes are one of the most fundamental principles of programming, managing and evaluating health sector in all countries. Information about death causes has long been used as a tool for observing the improvement in society’s health and for determining priorities of health measures (9). Recording and collecting information has long been used in industrial and developed countries based on current registration of death causes. Among some Latin American and Eastern Asian countries, just few could achieve a stable and statistically comparable system for death registration. In Iran, Ministry of Health, in association with Civil Status Registration Organization has taken a big step to determine mortality pattern and to observe its trend by registering causes of deaths according to ID Cards of deceased people. Recognizing mortality pattern and observing its trend will help us to determine health priorities, to allocate health resources and priorities in health sector, to eliminate main factors of premature deaths and to carry out epidemiological research (10).

## 2. Objective

The aim of this research was to determine avoidable deaths in order to recognize the performance of health care systems in providing the preventive and therapeutic cares.

## 3. Materials and Methods

Present descriptive-analytical study was carried out longitudinal. Research environment was all units affiliated to Kerman University of Medical Science and statistical universe was 20197 records of deceased cases which were selected through census method. In this study, avoidable deaths in Kerman between 2004 and 2010 were examined. Deaths were categorized to avoidable and unavoidable according to earlier work by Holland (11) after minor modification. It's worth mentioning that this disease list has been used in various studies in different continents and countries (12-14) to identify avoidable mortality (through treatment, prevention or both) and unavoidable mortality; in this research, it was used in the form of a checklist. This checklist consisted of the three sections: the first section collected data on demographic variables (age and gender), in the second part, data on avoidable mortality was collected and finally the third part was devoted to data on mortality. In this research, information of registered deaths was used. Data analysis was carried out using descriptive statistics and statistical tests (Chi square). To remove the effect of age in decreasing or increasing mortality, indirect age standardization method was used.

## 4. Results

Two thousand and one hundred –ninety seven records of deaths were examined. Most cases were male (59.17%) and their average age was 66.44. Findings showed that among all these cases (20197), 19987 cases were avoidable and 210 cases were unavoidable; 8697 cases included avoidable deaths thorough prevention (e.g. mortality due to heart diseases, lung cancer, drowning, falling, violence, hepatitis, burn, psychoactive drugs, AIDS, accidents), 11290 cases were avoidable through treatment (e.g. mortality due to breast cancer, colon and rectal cancer, stomach cancer, duodenal ulcer, diabetes, hypertension, tuberculosis, appendicitis, Hodgkin disease, uterus cancer, cervical uterine cancer, maternal deaths, birth insufficiency, lip and larynx cancer, gullet cancer, bladder cancer, urinary diseases, cerebral apoplexy). The 210 unavoidable cases included pancreas cancer, ovary cancer and prostate cancer (Table 1).

**Table 1.** Mortality in Terms of Disease Causes in Men and Women from 2004 to 2010 in Units Affiliated to Kerman University of Medical Science

Mortality in Terms of Disease Causes	Causes of Death			Death, No.
Avoidable Mortality	Age Limits	ICD-9	ICD-10	
**Breast cancer**	0-74	180	C53	152
**Colon and rectum cancer**	0-74	153-154	C18-C21	99
**Stomach cancer **	0-74	151	C16	107
**Peptic ulcer **	0-74	531-534	K25-K28	105
**Diabetes**	0-74	250	E10-E14	558
**Hypertensive disease **	0-74	402	I11	467
**Tuberculosis **	0-74	010-018،137	B90.A15-19	966
**Appendicitis **	0-74	540-543	K35-K38	120
**Hodgkin’s disease **	0-74	201	C81	19
**Cervix uteri and body of the uterus**	0-74	179,180,182	C54, C55C53	502
**Maternal deaths birth defects**	0-74	630,676, 237.70, 740-760	00-O99, H31.1, P00, P04, Q00-Q99	1496
**Lip, oral cavity and pharynx **	0-74	140-149	C00-C14	550
**Esophagus **	0-74	150	C15	27
**Bladder **	0-74	181	C67	70
**Urinary disease **	0-74	403, 580,589,591,593,592,594,598,7	I12, I13 N00-N09, N17-N19, N13, N20, N21, N35, N99.1	172
**Ischemic heart disease **	0-74	390-398,410-414	I20-I25 I01-I09	4434
**Stroke **	0-74			1446
**Preventable deaths**	0-74			
**Ischemic heart disease. All respiratory diseases (excluding pneumonia /influenza **	0-74	390-398,410-414	I20-I25 I01-I09	4434
**Hepatitis **	0-74	070	B15-B19	85
**Fires and burns **	0-74		X00-X09	129
**Illicit drug use disorders **	0-74			150
**HIV/AIDS **	0-74	070	B20-B24	30
**Road traffic injuries **	0-74	E810-E819	V01-04, V06, V09V80, V87V89, V99	1435
**Accidental poisoning falls **	0-74	E850- E869	X40-X49	28
**Drowning**	0-74	E896	W65-W74	4
**Violence **	0-74	E969- E960-	X85-Y09 , Y87.1	10
**Suicide and self-inflicted injuries **	0-74	E950,E959		1381
**Unavoidable mortality**				
**Pancreas**	0-74			40
**Prostate**	0-74	186	C62	162
**Ovarian**	0-74			8

**Figure 1.**
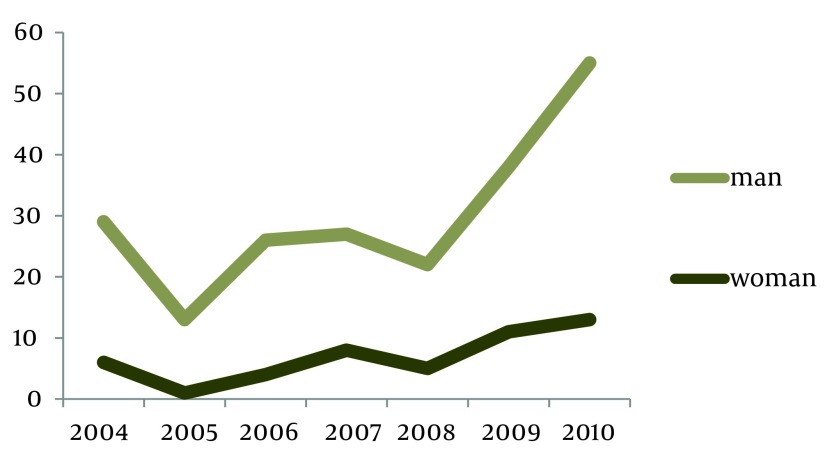
Study the Standardized Trend of Unavoidable Mortality Registered at University of Medical Science in Terms of Gender from 004 to 2010

**Figure 2.**
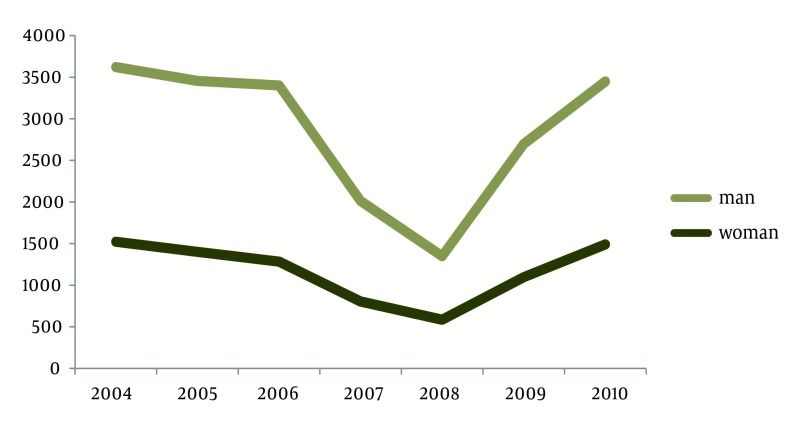
Study the Standardized Trend of Avoidable Mortality Registered at University of Medical Science in Terms of Gender from 2004 to 2010

Heart diseases were the first cause of avoidable death with 4434 cases. Maternal and fetal deaths, cerebral apoplexy and road accidents are the next causes of death in Kerman with 1496, 1446 and 1435 cases respectively. In Kerman, the lowest cases are related to appendicitis, asthma, AIDS, lip and larynx cancer, gullet cancer, bladder cancer, poisoning, and urinary diseases. In reviewing causes of death in Kerman, it was shown that number of mortality in all age groups was lower than the standard mortality in each age group (per ten thousand populations). The highest mortality rate was related to men aged more than 75. After examining mortality trend from 2004 to 2010, it was shown that avoidable mortality is increasing; it has increased by 22% in men and by 35% in women. It was also shown that unavoidable mortality has increased by 55% in men and by 18% in women (Figure 1 and 2). According to these results, avoidable mortality (through treatment and prevention) has increased in both genders.

According to the above graphs, avoidable mortality rate has decreased in 2008 in both men and women compared with other years.

## 5. Discussion

Avoidable mortality was 18.28% more in men than in women. In a study by Piers et al and in another study by Niti et al., it was shown that the highest mortality rate occurred in men (15, 16). It may be due to the fact that men are less willing to refer to treatment centers and that men are involved in different jobs and occupations outside the house and face more risks than women. A study by Nolet et al. showed that the highest mortality was related to risky health behaviors (smoking and no exercise). The reason is the difference among health systems in emphasizing treatment or health care. It was shown in this study that the highest mortality rate was related to people aged more than 75; it matches the study carried out by Nolte et al who showed that about half of the avoidable mortality (46.6) happens between 65 and 745. The reason is that this age group is exposed to more risk factors than other age groups. Results of this research showed that avoidable mortality increased by 22% in men and by 35% in women; they also showed that unavoidable mortality has increased by 55% in men and by 18% in women. However, a study by Burcine et al. in the Czech Republic revealed that avoidable mortality decreased by 40% in men and by 38% in women; unavoidable mortality decreased by 21% in men and 24% in women (17). It reflects less life expectancy between ages 0 and 75 and more in Kerman. It was observed in this study that heart diseases account for the most mortality (especially in men than in woman). In a study carried out by Chau et al., it was shown that cardiovascular diseases account for the highest mortality rate and it was more in women than in men (6). They match each other regarding high mortality rate of heart diseases. It may be as a result of changes in lifestyles and risky health behaviors (e.g. smoking, lack of exercise, social isolation, and lack of physical activity, violence and fatigue). It was revealed in this study that 98.86% of all deaths were avoidable; among them, 56.48% were thorough treatment and 43.52 were through prevention. Gaisauck et al. showed in his study that 72% of deaths were avoidable (46% through prevention and 54% through treatment) (18). Since avoidable mortality rate is high and since this trend increased sharply from 2004 to 2010, it can be concluded that in spite of progress and improvements in health system and presence of good specialists, health care system is not good and satisfactory in Kerman. In other words, studies about avoidable mortality trend revealed that this trend is increasing although it is not significant nowadays. However, if this trend continues, it will be very alarming for Iranian health authorities. Reduced avoidable mortality will increase life expectancy. Since more avoidable mortality happens in people more than 75, health system policy makers should pay more attention to health care in this vulnerable group, should recognize the causes of heart diseases which result in more deaths, and should take effective and preventive measures regarding changes in life style, long work hours, unsuitable mental-social environment in work place, social isolation and fatigue to reduce this mortality rate; they should also pay serious attention to treatment and preventive care in health system and evaluate efficiency and effectiveness of these services. This study showed that causes of most avoidable deaths are diseases that could be prevented through treatment and the other causes could be avoidable through prevention. These findings help us allocate some resources to health and treatment cares and invest in preventive and initial cares to prevent avoidable mortality through treatment and through prevention. Since investment in treatment is not economical, investment in preventive cares is more effective and productive in preventing mortality.
